# A survey on multimodal large language models

**DOI:** 10.1093/nsr/nwae403

**Published:** 2024-11-12

**Authors:** Shukang Yin, Chaoyou Fu, Sirui Zhao, Ke Li, Xing Sun, Tong Xu, Enhong Chen

**Affiliations:** School of Artificial Intelligence and Data Science, University of Science and Technology of China, Hefei 230026, China; State Key Laboratory for Novel Software Technology, Nanjing University, Nanjing 210023, China; School of Intelligence Science and Technology, Nanjing University, Suzhou 215163, China; School of Artificial Intelligence and Data Science, University of Science and Technology of China, Hefei 230026, China; Tencent YouTu Lab, Shanghai 200233, China; Tencent YouTu Lab, Shanghai 200233, China; School of Artificial Intelligence and Data Science, University of Science and Technology of China, Hefei 230026, China; School of Artificial Intelligence and Data Science, University of Science and Technology of China, Hefei 230026, China

**Keywords:** multimodal large language model, vision language model, large language model

## Abstract

Recently, the multimodal large language model (MLLM) represented by GPT-4V has been a new rising research hotspot, which uses powerful large language models (LLMs) as a brain to perform multimodal tasks. The surprising emergent capabilities of the MLLM, such as writing stories based on images and optical character recognition–free math reasoning, are rare in traditional multimodal methods, suggesting a potential path to artificial general intelligence. To this end, both academia and industry have endeavored to develop MLLMs that can compete with or even outperform GPT-4V, pushing the limit of research at a surprising speed. In this paper, we aim to trace and summarize the recent progress of MLLMs. First, we present the basic formulation of the MLLM and delineate its related concepts, including architecture, training strategy and data, as well as evaluation. Then, we introduce research topics about how MLLMs can be extended to support more granularity, modalities, languages and scenarios. We continue with multimodal hallucination and extended techniques, including multimodal in-context learning, multimodal chain of thought and LLM-aided visual reasoning. To conclude the paper, we discuss existing challenges and point out promising research directions.

## Teaser

This paper presents the first survey on Multimodal Large Language Models (MLLMs), highlighting their potential as a path to Artificial General Intelligence (AGI).

## INTRODUCTION

Recent years have seen remarkable progress in large language models (LLMs) [1,2]. By scaling up data size and model size, these LLMs raise extraordinary emergent abilities, typically including instruction following [3], in-context learning (ICL) [4] and chain of thought (CoT) [5]. Although LLMs have demonstrated surprising zero/few-shot reasoning performance on most natural language processing (NLP) tasks [6] and even complex real-life applications [7–9], they are inherently ‘blind’ to vision since they can only understand discrete text. At the same time, large vision models (LVMs) can see clearly [10,11], but commonly lag in reasoning.

In light of this complementarity, an LLM and LVM run towards each other, leading to the new field of the multimodal large language model (MLLM). Formally, it refers to the LLM-based model with the ability to receive, reason and output with multimodal information. Prior to the MLLM, there have been a lot of works devoted to multimodality, which can be divided into discriminative [12,13] and generative [14,15] paradigms. Contrastive language-image pre-training (CLIP) [12], as a representative of the former, projects visual and textual information into a unified representation space, building a bridge for downstream multimodal tasks. In contrast, one for all (OFA) [14] is a representative of the latter, which unifies multimodal tasks in a sequence-to-sequence manner. The MLLM can be classified as the latter according to the sequence operation, but it manifests two distinct traits compared with its traditional counterparts.

- The MLLM is based on an LLM with billion-scale parameters, which is not available in previous models.
- The MLLM uses new training paradigms to unleash its full potential, such as using multimodal instruction tuning [16] to encourage the model to follow new instructions.

Armed with the two traits, the MLLM exhibits new capabilities, such as writing website code based on images [17], understanding the deep meaning of a meme [18] and optical character recognition– (OCR) free math reasoning [19].

Ever since the release of GPT-4 [20], there has been a research frenzy over MLLMs because of the amazing multimodal examples it shows. Rapid development is fueled by efforts from both academia and industry. Preliminary research on MLLMs focuses on text content generation grounded in text prompts and image [16]/video [21,22]/audio [23]. Subsequent works have expanded the capabilities or the usage scenarios, including:

- better granularity support—finer control on user prompts is developed to support specifying regions through boxes [24] or a certain object through a click [25];
- enhanced support on input and output modalities [26,27], such as image, video, audio and point cloud;
- improved language support—efforts have been made to extend the success of MLLMs to other languages (e.g. Chinese) with relatively limited training corpus [28];
- extension to more realms and usage scenarios—some studies transfer the strong capabilities of MLLMs to other domains, such as medical image understanding [29] and document parsing [30].

**Figure 1.**
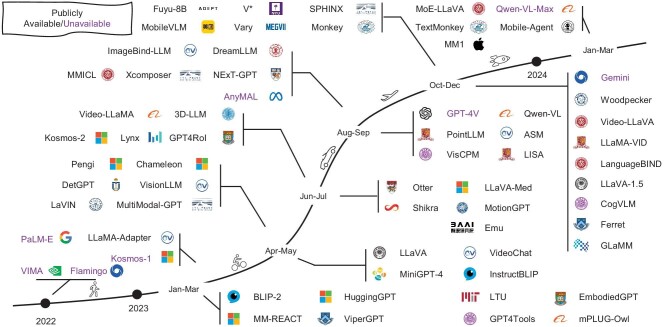
A timeline of representative MLLMs. We are witnessing rapid growth in this field. More works can be found on our released GitHub page, which is updated daily.

Moreover, multimodal agents are developed to assist in real-world interaction, e.g. embodied agents [31] and graphical user interface (GUI) agents [32]. An MLLM timeline is illustrated in Fig. 1.

In view of such rapid progress and the promising results of this field, we have written this survey to provide researchers with a grasp of the basic idea, main method and current progress in MLLMs. Note that we mainly focus on visual and language modalities, but also include works involving other modalities like video and audio. Specifically, we cover the most important aspects of MLLMs with corresponding summaries and have opened a GitHub page that will be updated in real time. To the best of our knowledge, this is the first survey on the MLLM.

The survey is structured as follows. We start with a comprehensive review of the essential aspects of MLLMs, including the mainstream architecture, a full recipe for the training strategy and data, and common practices for performance evaluation. Then, we delve into a deeper discussion of some important topics about MLLMs, each focusing on one of the following main problems. (i) What aspects can be further improved or extended? (ii) How can we relieve the multimodal hallucination issue? The survey continues with the introduction of three key techniques, each specialized in a specific scenario. Multimodal in-context learning is an effective technique commonly used at the inference stage to boost few-shot performance. Another important technique is multimodal chain of thought, which is typically used in complex reasoning tasks. Afterward, we delineate general ideas for developing LLM-based systems to solve composite reasoning tasks or to address common user queries. We conclude our survey with a summary and potential research directions.

## ARCHITECTURE

**Figure 2.**
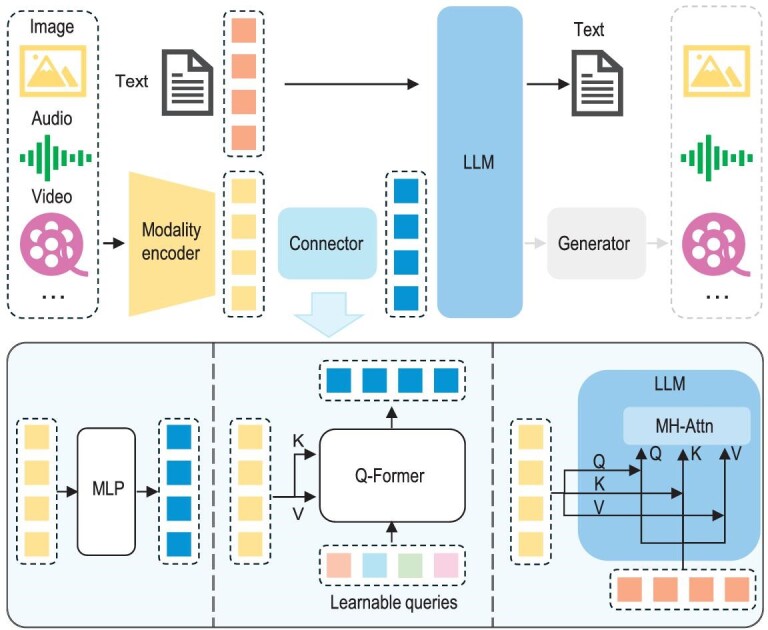
An illustration of typical MLLM architecture. It includes an encoder, a connector and an LLM. An optional generator can be attached to the LLM to generate more modalities besides text. The encoder takes in images, audios or videos and outputs features, which are processed by the connector so that the LLM can better understand. There are broadly three types of connector: projection-based, query-based and fusion-based connectors. The former two types adopt token-level fusion, processing features into tokens to be sent along with text tokens, while the last type enables a feature-level fusion inside the LLM.

A typical MLLM can be abstracted into three modules: a pre-trained modality encoder, a pre-trained LLM and a modality interface to connect them. Drawing an analogy to humans, modality encoders such as image/audio encoders are human eyes/ears that receive and pre-process optical/acoustic signals, while LLMs are like human brains that understand and reason with the processed signals. In between, the modality interface serves to align different modalities. Some MLLMs also include a generator to output other modalities apart from text. A diagram of the architecture is plotted in Fig. 2. In this section, we introduce each module in sequence.

### Modality encoder

The encoders compress raw information, such as images or audio, into a more compact representation. Rather than training from scratch, a common approach is to use a pre-trained encoder that has been aligned to other modalities. For example, CLIP [12] incorporates a visual encoder semantically aligned with the text through large-scale pre-training on image-text pairs. Therefore, it is more practical to utilize such pre-aligned encoders to align with LLMs through alignment pre-training.

Commonly used image encoders are summarized in Table 1. Apart from vanilla CLIP image encoders [12], some works also explore using other variants. For example, MiniGPT-4 [17] adopts an EVA-CLIP [36] (ViT-G/14) encoder, which is trained with improved training techniques. Osprey [25] introduces a convolution-based ConvNext-L encoder [33] to utilize higher resolution and multi-level features. Some works also explore an encoder-free architecture. For instance, the image patches of Fuyu-8b [37] are directly projected before sending to LLMs. With this design, the model naturally supports flexible input image resolution.

**Table 1.** A summary of commonly used image encoders.

Variants	Pre-training corpus	Resolution	Samples (B)	Parameter size (M)
OpenCLIP-ConvNext-L [33]	LAION-2B	320	29	197.4
CLIP-ViT-L/14 [12]	OpenAI’s WIT	224/336	13	304.0
EVA-CLIP-ViT-G/14 [34]	LAION-2B,COYO-700M	224	11	1000.0
OpenCLIP-ViT-G/14 [33]	LAION-2B	224	34	1012.7
OpenCLIP-ViT-bigG/14 [33]	LAION-2B	224	34	1844.9
InternViT-6B [35]	Multiple datasets	448	–	5540.0

When choosing encoders, one often considers factors such as resolution, parameter size and pre-training corpus. Notably, many works have empirically verified that using higher resolution can achieve remarkable performance gains [28,38]. The approaches for scaling up input resolution can be categorized into direct scaling and patch-division methods. The direct scaling method inputs images of higher resolutions to the encoder, which often involves further tuning the encoder [28] or replacing a pre-trained encoder with higher resolution [39]. Similarly, CogAgent [32] uses a dual-encoder mechanism, where two encoders process high- and low-resolution images, respectively. High-resolution features are injected into the low-resolution branch through cross-attention. Patch-division methods cut a high-resolution image into patches and reuse the low-resolution encoder. For example, Monkey [38] and SPHINX [40] divide a large image into smaller patches and send sub-images together with a downsampled high-resolution image to the image encoder, where the sub-images and the low-resolution image capture local and global features, respectively. In contrast, parameter size and training data composition are of less importance compared with input resolution, as found by empirical studies [41].

Similar encoders are also available for other modalities. For example, Pengi [23] uses the CLAP [42] model as the audio encoder. ImageBind-LLM [26] uses the ImageBind [43] encoder, which supports encoding image, text, audio, depth, thermal and inertial measurement unit data. Equipped with the strong encoder, the ImageBind-LLM can respond to the input of various modalities.

### Pre-trained LLM

Instead of training an LLM from scratch, it is more efficient and practical to start with a pre-trained one. Through tremendous pre-training on the web corpus, LLMs have been embedded with rich world knowledge, and demonstrate strong generalization and reasoning capabilities.

We summarize the commonly used and publicly available LLMs in Table 2. Notably, most LLMs fall in the causal decoder category, following GPT-3 [4]. Among them, Flan-T5 [44] series are relatively early LLMs used in works like BLIP-2 [50] and InstructBLIP [51]. LLaMA series [45] and the Vicuna family [46] are representative open-sourced LLMs that have attracted much academic attention. Since the two LLMs are mainly pre-trained on the English corpus, they are limited in multi-language support, such as Chinese. In contrast, Qwen [48] is a bilingual LLM with Chinese and English support.

**Table 2.** A summary of commonly used open-sourced LLMs. En, Zh, Fr and De stand for English, Chinese, French and German, respectively.

	Release	Pre-train	Parameter size	Language	
Model	date	data scale	(B)	support	Architecture
Flan-T5-XL/XXL [44]	Oct. 2022	–	3/11	En, Fr, De	Encoder decoder
LLaMA [45]	Feb. 2023	1.4T tokens	7/13/33/65	En	Causal decoder
Vicuna [46]	March 2023	1.4T tokens	7/13/33	En	Causal decoder
LLaMA-2 [47]	July 2023	2T tokens	7/13/70	En	Causal decoder
Qwen [48]	Sept. 2023	3T tokens	1.8/7/14/72	En, Zh	Causal decoder
LLaMA-3 [49]	April 2024	15T tokens	8/70/405	En, Fr, De, etc.	Causal decoder

It should be noted that scaling up the parameter size of LLMs also brings additional gains, similar to the case of increasing input resolution. Specifically, Liu *et al.* [39,52] found that simply scaling up the LLM from 7B to 13B brings comprehensive improvement on various benchmarks. Furthermore, when using a 34B LLM, the model shows emergent zero-shot Chinese capability, given that only English multimodal data are used during training. Lu *et al.* [53] observed a similar phenomenon by scaling up LLMs from 13B to 35B and 65B/70B, where the larger model size brings consistent gains on benchmarks specifically designed for MLLMs. Some works instead use smaller LLMs to facilitate deployment on mobile devices. For example, MobileVLM series [54] use downscaled LLaMA [45] to enable efficient inference on mobile processors.

Recently, explorations of the mixture-of-experts (MoE) architecture for LLMs have garnered rising attention [55]. Compared with dense models, the sparse architecture enables scaling up the total parameter size without increasing the computational cost, by selective activation of the parameters. Empirically, MM1 [41] and MoE-LLaVA [56] find that MoE implementation achieves better performance than the dense counterpart on almost all the benchmarks.

### Modality interface

Since LLMs can only perceive text, bridging the gap between natural language and other modalities is necessary. Nevertheless, it would be costly to train from scratch a large multimodal model in an end-to-end manner. A more practical way is to introduce a learnable connector between the pre-trained visual encoder and LLM. The other approach is to translate images into languages with the help of expert models, and then send the language to the LLM.

#### Learnable connector

The learnable connector is responsible for bridging the gap between different modalities. Specifically, the module projects information into the space that the LLM can understand efficiently. Based on how multimodal information is fused, there are broadly two ways to implement such interfaces: token-level and feature-level fusion for different modalities.

For token-level fusion, features output from encoders are transformed into tokens and concatenated with text tokens before being sent into LLMs. A common solution is to leverage a group of learnable query tokens to extract information in a query-based manner [57], which was first implemented in BLIP-2 [50], and subsequently inherited by a variety of works [22,51]. Such Q-Former-style approaches compress visual tokens into a smaller number of representation vectors. In contrast, some methods simply use an MLP-based interface to bridge the modality gap [16]. For example, LLaVA series adopt an MLP [16,39] to project visual tokens and align the feature dimension with word embeddings. BLIVA [58] adopts an ensemble of MLP-based and Q-Former-based connectors to enhance performance in text-rich scenarios.

As another line, feature-level fusion inserts extra modules that enable deep interaction and fusion between text features and visual features. For example, Flamingo [59] inserts extra cross-attention layers between frozen transformer layers of LLMs, thereby augmenting language features with external visual cues. Similarly, CogVLM [60] plugs in a visual expert module in each transformer layer to enable dual interaction and fusion between vision and language features. For better performance, the QKV weight matrix of the introduced module is initialized from the pre-trained LLM. Likewise, LLaMA-Adapter [61] introduces learnable prompts into transformer layers. These prompts are first embedded with visual knowledge and then concatenated with text features as prefixes.

On a related note, MM1 [41] has conducted ablation studies on the design choices of the connector and found that, for token-level fusion, the type of modality adapter is far less important than the number of visual tokens and input resolution. Nevertheless, Zeng *et al.* [62] compared the performance of token- and feature-level fusion, and empirically revealed that the token-level fusion variant performs better in terms of VQA benchmarks. Regarding the performance gap, the authors suggested that cross-attention models might require a more complicated hyper-parameter searching process to achieve comparable performance.

In terms of parameter size, learnable interfaces generally comprise a small portion compared with encoders and LLMs. Take Qwen-VL [28] as an example; the parameter size of the Q-Former is about 0.08B, accounting for less than 1% of the whole parameters, while the encoder and the LLM account for about 19.8% (1.9B) and 80.2% (7.7B), respectively.

#### Expert model

Apart from the learnable interface, using expert models, such as an image captioning model, is also a feasible way to bridge the modality gap [63]. The basic idea is to convert multimodal inputs into languages without training. In this way, LLMs can understand multimodality by the converted languages. For example, VideoChat-Text [21] uses pre-trained vision models to extract visual information such as actions and enriches the descriptions using a speech recognition model. Though using expert models is straightforward, it may not be as flexible as adopting a learnable interface. The conversion of foreign modalities into text would cause information loss. For example, transforming videos into textual descriptions distorts spatial-temporal relationships [21].

## TRAINING STRATEGY AND DATA

A full-fledged MLLM undergoes three stages of training: pre-training, instruction tuning and alignment tuning. Each phase of training requires different types of data and fulfills different objectives. In this section, we discuss training objectives, as well as data collection and characteristics for each training stage.

### Pre-training

#### Training detail

As the first training stage, pre-training mainly aims to align different modalities and learn multimodal world knowledge. The pre-training stage generally entails large-scale text-paired data, e.g. caption data. Typically, the caption pairs describe images/audio/videos in natural language.

**Scheme 1.**
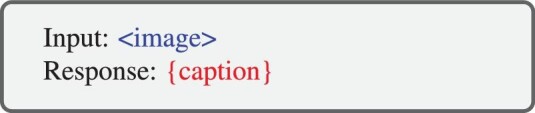
A simplified template to structure the caption data. {$< $image$> $} is the placeholder for the visual tokens, and {caption} is the caption for the image. Note that only the part marked in red is used for loss calculation.

Here, we consider a common scenario where MLLMs are trained to align vision with text. As illustrated in Scheme 1, given an image, the model is trained to autoregressively predict the caption of the image, following a standard cross-entropy loss. A common approach for pre-training is to freeze pre-trained modules (e.g. visual encoders and LLMs) and train a learnable interface [16]. The idea is to align different modalities without losing pre-trained knowledge. Some methods [28] also unfreeze more modules (e.g. the visual encoder) to enable more trainable parameters for alignment. It should be noted that the training scheme is closely related to data quality. For short and noisy caption data, using lower resolution (e.g. 224 pixels) can speed up the training process, while for longer and cleaner data, it is better to utilize higher resolutions (e.g. 448 pixels or higher) to mitigate hallucinations. Besides, ShareGPT4V [64] finds that, with high-quality caption data in the pre-training stage, unlocking the vision encoder promotes better alignment.

#### Data

Pre-training data mainly serve two purposes: aligning different modalities and providing world knowledge. The pre-training corpora can be divided into coarse-grained and fine-grained data according to granularities, which we will introduce sequentially. We summarize commonly used pre-training datasets in Table 3.

**Table 3.** Common datasets used for pre-training.

Dataset	Samples	Date
**Coarse-grained image text**		
CC-3M [65]	3.3M	2018
CC-12M [66]	12.4M	2020
SBU Captions [67]	1M	2011
LAION-5B [68]	5.9B	Mar. 2022
LAION-2B [68]	2.3B	Mar. 2022
LAION-COCO [69]	600M	Sep. 2022
COYO-700M [71]	747M	Aug. 2022
**Fine-grained image text**		
ShareGPT4V-PT [64]	1.2M	Nov. 2023
LVIS-Instruct4V [72]	111K	Nov. 2023
ALLaVA [73]	709K	Feb. 2024
**Video text**		
MSR-VTT [74]	200K	2016
**Audio text**		
WavCaps [75]	24K	Mar. 2023

Coarse-grained caption data share some typical traits in common. (i) The data volume is large since samples are generally sourced from the internet. (ii) Because of the web-scrawled nature, the captions are usually short and noisy since they originate from the alt-text of the web images. These data can be cleaned and filtered via automatic tools, for example using the CLIP [12] model to filter out image-text pairs whose similarities are lower than a predefined threshold. In what follows, we introduce some representative coarse-grained datasets.

*CC Series.* CC-3M [65] is a web-scale caption dataset of 3.3M image-caption pairs, where the raw descriptions are derived from alt-text associated with images. The authors designed a complicated pipeline to clean data. For images, those with inappropriate content or aspect ratio are filtered. For text, NLP tools are used to obtain text annotations, with samples filtered according to the designed heuristics. For image-text pairs, images are assigned labels via classifiers. If text annotations do not overlap with image labels, the corresponding samples are dropped.

CC-12M [66] is a following work of CC-3M and contains 12.4M image-caption pairs. Compared with the previous work, CC-12M relaxes and simplifies the data-collection pipeline, thus collecting more data.

*SBU Captions [67].* This is a captioned photo dataset containing 1M image-text pairs, with images and descriptions sourced from Flickr. Specifically, an initial set of images is acquired by querying the Flickr website with a large number of query terms. The descriptions attached to the images thus serve as captions. Then, to ensure that descriptions are relevant to the images, the retained images fulfill the following requirements:

- descriptions of the images are of satisfactory length, decided by observation;
- captions should contain at least two words in the predefined term lists and a propositional word (e.g. ‘on’, ‘under’) that suggests spatial relationships.

*LAION.* These series are large web-scale datasets, with images scrawled from the internet and associated alt-text as captions. To filter the image-text pairs, the following steps are performed:

- text with short lengths or images with too small or too big sizes are dropped;
- image deduplication is performed based on the URL;
- CLIP [12] embeddings for images and text are extracted, and the embeddings are used to drop possibly illegal content and image-text pairs with low cosine similarity between embeddings.

Here we offer a brief summary of some typical variants.

- *LAION-5B [68].* This variant is a research-purpose dataset of 5.85B image-text pairs. The dataset is multilingual with a 2B English subset.
- *LAION-COCO [69].* This variant contains 600M images extracted from the English subset of LAION-5B. The captions are synthetic, using BLIP [70] to generate various image captions and using CLIP [12] to pick the best fit.

*COYO-700M [71].* This dataset contains 747M image-text pairs, which are extracted from CommonCrawl. In terms of data filtering, the authors designed the following strategies to filter out data samples. For images, those with inappropriate size, content, format or aspect ratio are filtered. Moreover, the images are filtered based on the pHash value to remove images overlapped with public datasets such as ImageNet and MS-COCO. For text, only English text with satisfactory length, noun forms and appropriate words are saved. Whitespace before and after the sentence will be removed, and consecutive whitespace characters will be replaced with a single whitespace. Moreover, text appearing more than 10 times (e.g. ‘image for’) will be dropped. For image-text pairs, duplicated samples are removed based on the (pHash, text) tuple.

Recently, more works [64,73] have explored generating high-quality fine-grained data through prompting strong MLLMs (e.g. GPT-4V). Compared with coarse-grained data, these data generally contain longer and more accurate descriptions of the images, thus enabling finer-grained alignment between image and text modalities. However, since the approach generally requires calling commercial-use MLLMs, the cost is higher, and the data volume is smaller. Notably, ShareGPT4V [64] strikes a balance by first training a captioner with GPT-4V-generated 100K data, then scaling up the data volume to 1.2M using the pre-trained captioner.

### Instruction tuning

#### Introduction

Instruction refers to the description of tasks. Intuitively, instruction tuning aims to teach models to better understand the instructions from users and fulfill the demanded tasks. Tuning in this way, LLMs can generalize to unseen tasks by following new instructions, thus boosting zero-shot performance. This simple yet effective idea has sparked the success of subsequent NLP works, such as ChatGPT [77] and InstructGPT [78].

**Figure 3.**
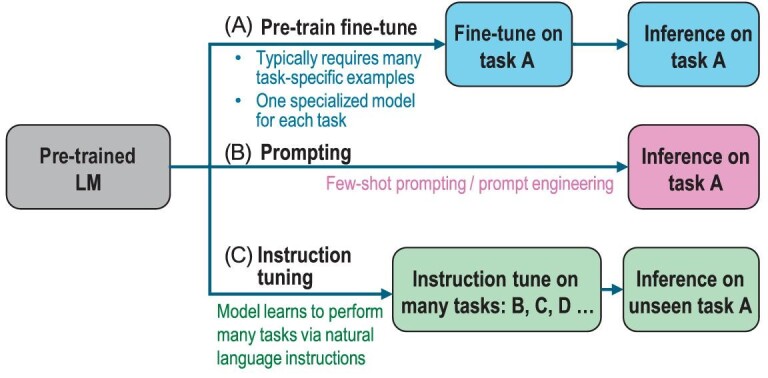
Comparison of three typical learning paradigms, adapted from [76].

The comparisons between instruction tuning and related typical learning paradigms are illustrated in Fig. 3. The supervised fine-tuning approach usually requires a large amount of task-specific data to train a task-specific model. The prompting approach reduces the reliance on large-scale data and can fulfill a specialized task via prompt engineering. In such a case, though the few-shot performance has been improved, the zero-shot performance is still quite average [4]. Differently, instruction tuning learns how to generalize to unseen tasks rather than fitting specific tasks like the two counterparts. Moreover, instruction tuning is highly related to multi-task prompting [79] and learning [80].

In this section, we delineate the format of instruction samples, training objectives, typical ways to gather instruction data and corresponding commonly used datasets.

#### Training detail

**Scheme 2.**
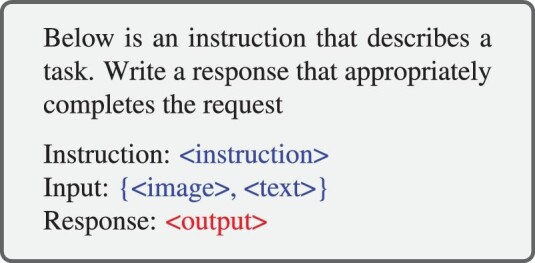
A simplified template to structure the multimodal instruction data. $< $instruction$> $ is a textual description of the task. {$< $image$> $, $< $text$> $} and $< $output$> $ are the input and output from the data sample. Note that $< $text$> $ in the input may be missed for some datasets; for example, image caption datasets merely have $< $image$> $. The example is adapted from [81].

A multimodal instruction sample often includes an optional instruction and an input-output pair. The instruction is typically a natural language sentence describing the task, such as ‘Describe the image in detail.’ The input can be an image-text pair like the VQA task [82] or only an image like the image caption task [83]. The output is the answer to the instruction conditioned on the input. The instruction template is flexible and subject to manual designs [21], as exemplified in Scheme 2. Note that the instruction template can also be generalized to the case of multi-round human-agent conversations [16,81].

Formally, a multimodal instruction sample can be denoted in a triplet form, i.e. $(\mathcal {I}, \mathcal {M}, \mathcal {R})$, where $\mathcal {I}, \mathcal {M}, \mathcal {R}$ represent the instruction, the multimodal input and the ground-truth response, respectively. The MLLM predicts an answer given the instruction and the multimodal input:


(1) \begin{eqnarray*}
\mathcal {A} = f(\mathcal {I}, \mathcal {M}; \theta ).
\end{eqnarray*}

Here, $\mathcal {A}$ denotes the predicted answer, and $\theta$ are the parameters of the model. The training objective is typically the original auto-regressive objective used to train LLMs [16], based on which the MLLM is encouraged to predict the next token of the response sequentially:


(2) \begin{eqnarray*}
\mathcal {L}(\theta ) = -\sum _{i=1}^{N} \log p(\mathcal {R}_i\mid \mathcal {I}, \mathcal {R}_{< i}; \theta )
\end{eqnarray*}

with *N* the length of the ground truth.

#### Data collection

Since instruction data are more flexible in formats and varied in task formulations, it is usually trickier and more costly to collect data samples. In this section, we summarize three typical ways to harvest instruction data at scale: data adaptation, self-instruction and data mixture.

**Scheme 3.**
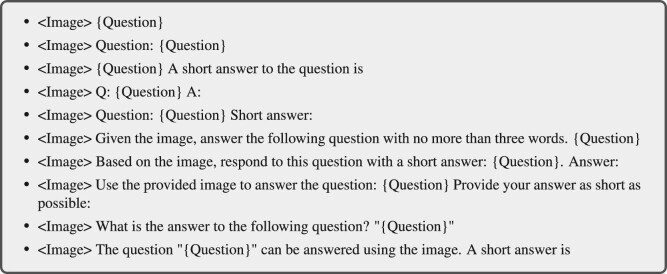
Instruction templates for VQA datasets, cited from [51]. $< $Image$> $ and {Question} are the image and the question in the original VQA datasets, respectively.

*Data adaptation.* Task-specific datasets are rich sources of high-quality data. Hence, abundant works [51,84] have utilized existing high-quality datasets to construct instruction-formatted datasets. Take the transformation of VQA datasets as an example; the original sample is an input-out pair where the input comprises an image and a natural language question, and the output is the textual answer to the question conditioned on the image. The input-output pairs of these datasets could naturally comprise the multimodal input and response of the instruction sample. The instructions, i.e. the descriptions of the tasks, can either derive from manual design or from semi-automatic generation aided by GPT. Specifically, some works [17] handcraft a pool of candidate instructions and sample one of them during training. We offer an example of instruction templates for the VQA datasets in Scheme 3. The other works manually design some seed instructions and use these to prompt GPT to generate more [21].

Note that, since the answers of existing VQA and caption datasets are usually concise, directly using these datasets for instruction tuning may limit the output length of MLLMs. There are two common strategies to tackle this problem. The first strategy is to specify the corresponding requirements explicitly in the instructions. For example, ChatBridge [85] explicitly declares *short* and *brief* for short-answer data. The second strategy is to extend the length of existing answers [86]. For example, M$^3$IT [86] proposes to rephrase the original answer by prompting ChatGPT with the original question, answer and contextual information of the image (e.g. caption and text extracted through OCR).

*Self-instruction.* Although existing multi-task datasets can contribute a rich source of data, they usually do not meet human needs well in real-world scenarios, such as multiple-round conversations. To tackle this issue, some works collect samples through self-instruction [89], which utilizes LLMs to generate textual instruction-following data using a few hand-annotated samples. Specifically, some instruction-following samples are handcrafted as demonstrations, after which ChatGPT/GPT-4 is prompted to generate more instruction samples with the demonstrations as guidance. LLaVA [16] extends the approach to the multimodal field by translating images into text of captions and bounding boxes, and prompting text-only GPT-4 to generate new data with the guidance of requirements and demonstrations. In this way, a multimodal instruction dataset is constructed, called LLaVA-Instruct-150k. Following this idea, subsequent works such as MiniGPT-4 [17] and GPT4Tools [90] develop different datasets catering to different needs. Recently, with the release of the more powerful multimodal model GPT-4V, many works have adopted GPT-4V to generate data of higher quality, as exemplified by LVIS-Instruct4V [72] and ALLaVA [73]. We summarize the popular datasets generated through self-instruction in Table 4. It should be noted that this paradigm highly relies on advanced yet close-sourced models, which can be expensive for data scaling. This approach might be partially due to the limited capabilities of early models. Future research can explore capitalizing on open-sourced models to generate high-quality instruction data.

**Table 4.** A summary of popular datasets generated by self-instruction. For input/output modalities, I denotes image, T denotes text, V denotes video, A denotes audio. For data composition, M-T and S-T denote multi-turn and single-turn, respectively.

Dataset	Sample	Modality	Source	Composition
LLaVA-Instruct [16]	158K	I + T $\rightarrow$ T	MS-COCO	23K caption + 58K M-T QA + 77K reasoning
LVIS-Instruct [72]	220K	I + T $\rightarrow$ T	LVIS	110K caption + 110K M-T QA
ALLaVA [73]	1.4M	I + T $\rightarrow$ T	VFlan, LAION	709K caption + 709K S-T QA
Video-ChatGPT [87]	100K	V + T $\rightarrow$ T	ActivityNet	7K description + 4K M-T QA
VideoChat [21]	11K	V+T $\rightarrow$ T	WebVid	description + summarization + creation
Clotho-Detail [88]	3.9K	A + T $\rightarrow$ T	Clotho	caption

*Data mixture.* Apart from the multimodal instruction data, language-only user-assistant conversation data can also be used to improve conversational proficiencies and instruction-following abilities [91]. LaVIN [91] directly constructs a minibatch by randomly sampling from both language-only and multimodal data. MultiInstruct [84] probes different strategies for training with a fusion of single-modal and multimodal data, including mixed instruction tuning (combine both types of data and randomly shuffle) and sequential instruction tuning (text data followed by multimodal data).

#### Data quality

Recent research has revealed that the data quality of instruction-tuning samples is no less important than quantity. Lynx [62] finds that models pre-trained on large-scale but noisy image-text pairs do not perform as well as models pre-trained with smaller but cleaner datasets. Similarly, Wei *et al.* [92] found that less instruction-tuning data with higher quality can achieve better performance. For data filtering, the work proposes some metrics to evaluate data quality and, correspondingly, a method to automatically filter out inferior vision-language data. Here we discuss two important aspects of data quality.

*Prompt diversity.* The diversity of instructions has been found to be critical for model performance. Lynx [62] empirically verifies that diverse prompts help improve model performance and generalization ability.

*Task coverage.* In terms of tasks involved in training data, Du *et al.* [93] performed an empirical study and found that the visual reasoning task is superior to captioning and QA tasks for boosting model performance. Moreover, the study suggests that more complex instructions are better than increasing task diversity and incorporating fine-grained spatial annotations.

### Alignment tuning

#### Introduction

Alignment tuning is more often used in scenarios where models need to be aligned with specific human preferences, e.g. response with fewer hallucinations. Currently, reinforcement learning with human feedback (RLHF) and direct preference optimization (DPO) are two main techniques for alignment tuning. In this section, we introduce the main ideas of the two techniques in sequence, offer some examples of how they are utilized in addressing practical problems and, finally, give a compilation of the related datasets.

#### Training detail

*RLHF [94,95].* This technique aims to utilize reinforcement learning algorithms to align LLMs with human preferences, with human annotations as supervision in the training loop. As exemplified in InstructGPT [78], RLHF incorporates three key steps.

- *Supervised fine-tuning.* This step aims to fine-tune a pre-trained model to present the preliminary desired output behavior. The fine-tuned model in the RLHF setting is called a *policy model*. Note that this step might be skipped since the supervised policy model $\pi ^{\text{SFT}}$ can be initialized from an instruction-tuned model.
- *Reward modeling.* A *reward model* is trained using preference pairs in this step. Given a multimodal prompt (e.g. image and text) *x* and a response pair $(y_w, y_l)$, the reward model $r_\theta$ learns to give a higher reward to the preferred response $y_w$, and vice versa for $y_l$, with the objective
  (3) \begin{eqnarray*}
\mathcal {L}(\theta )& =& -\mathbb {E}_{(x,y_w,y_l)\sim \mathcal {D}} \log \sigma [r_\theta (x, y_w) \\
&& -\, r_\theta (x, y_l) ],
\end{eqnarray*}
  where $\mathcal {D}=\lbrace (x,y_w,y_l)\rbrace$ is the comparison dataset labeled by human annotators. In practice, the reward model $r_\theta$ shares a similar structure with the policy model.
- *Reinforcement learning.* In this step, the proximal policy optimization (PPO) algorithm is adopted to optimize the RL policy model $\pi ^{\text{RL}}_\phi$. A per-token KL penalty is often added to the training objective to avoid deviating too far from the original policy [78], resulting in the objective
  (4) \begin{eqnarray*}
\mathcal {L}(\phi ) &=& -\mathbb {E}_{x\sim \mathcal {D}, y\sim \pi ^{\rm RL}_{\phi }(y\mid x)} \Big[ r_\theta (x,y) - \beta \cdot \mathbb {D}_{\rm KL} \\
&&\times \, \Big\lbrace \pi ^{\rm RL}_{\phi }(y\mid x) \Vert \pi ^{\rm REF}(y\mid x)\Big\rbrace\Big],\!\\
\end{eqnarray*}
  where $\beta$ is the coefficient for the KL penalty term. Typically, both the RL policy $\pi ^{\text{RL}}_\phi$ and the reference model $\pi ^{\text{REF}}$ are initialized from the supervised model $\pi ^{\text{SFT}}$. The obtained RL policy model is expected to align with human preferences through this tuning process.

Researchers have explored using the RLHF techniques for better multimodal alignment. For example, LLaVA-RLHF [96] collects human preference data and tunes a model with fewer hallucinations based on LLaVA [16].

*DPO [97].* This technique learns from human preference labels, utilizing a simple binary classification loss. Compared with the PPO-based RLHF algorithm, DPO is exempt from learning an explicit reward model, thus simplifying the whole pipeline to two steps: human preference data collection and preference learning. The learning objective for the algorithm is


(5) \begin{eqnarray*}
\mathcal {L}(\phi ) &=& -\mathbb {E}_{(x,y_w,y_l)\sim \mathcal {D}} \bigg [ \log \sigma \bigg ( \beta \log \frac{\pi _\phi ^{\text{RL}}(y_w\!\mid\! x)}{\pi ^{\text{REF}}(y_w\!\mid\! x)} \\
&& -\, \beta \log \frac{\pi _\phi ^{\text{RL}}(y_l\!\mid\! x)}{\pi ^{\text{REF}}(y_l\!\mid\! x)} \bigg )\bigg ].
\end{eqnarray*}

RLHF-V [98] collects fine-grained (segment-level) preference data pairs by correcting hallucinations in the model response and uses the obtained data to perform dense DPO. Silkie [99] instead collects preference data via prompting GPT-4V and distills the preference supervision into an instruction-tuned model through DPO.

#### Data

The gist of data collection for alignment tuning is to collect feedback for model responses, i.e. to decide which response is better. It is generally more expensive to collect such data, and the amount of data used for this phase is typically even less than that used in previous stages. In this part, we introduce some datasets and summarize them in Table 5.

**Table 5.** A summary of datasets for alignment tuning. For input/output modalities, I denotes image and T denotes text.

Dataset	Sample	Modality	Source
LLaVA-RLHF [96]	10K	I + T $\rightarrow$ T	Human
RLHF-V [98]	5.7K	I + T $\rightarrow$ T	Human
VLFeedback [99]	380K	I + T $\rightarrow$ T	GPT-4V

*LLaVA-RLHF [96].* This dataset contains 10K preference pairs collected from human feedback in terms of honesty and helpfulness. It mainly serves to reduce hallucinations.

*RLHF-V [98].* This dataset has 5.7K fine-grained human feedback data collected by performing segment-level hallucination corrections.

*VLFeedback [99].* This dataset utilizes AI to provide feedback on model responses. It contains more than 380K comparison pairs scored by GPT-4V in terms of helpfulness, faithfulness and ethical concerns.

## Evaluation

Evaluation is an essential part of developing MLLMs since it provides feedback for model optimization and helps to compare the performance of different models. Compared with evaluation methods of traditional multimodal models, the evaluation of MLLMs exhibits several new traits. (1) Since MLLMs are generally versatile, it is important to evaluate MLLMs comprehensively. (2) MLLMs exhibit many emergent capabilities that require special attention (e.g. OCR-free math reasoning) and thus require new evaluation schemes. The evaluation of MLLMs can be broadly categorized into two types according to the question genres: closed-set and open-set evaluation. Closed-set evaluation often involves task-specific benchmarks and more comprehensive benchmarks specifically designed for the MLLM, where answers are limited to predefined sets. Open-set evaluation typically includes manual scoring, GPT scoring and case study.

### Closed set

Closed-set questions refer to a type of question where the possible answer options are predefined and limited to a finite set. The evaluation is usually performed on task-specific datasets. In this case, the responses can be naturally judged by benchmark metrics. For example, InstructBLIP [51] reports the accuracy on ScienceQA [100], as well as the CIDEr score [101] on NoCaps [102]. The evaluation setting is typically zero shot [51,84] or fine-tuning [29,51]. The first setting often selects a wide range of datasets covering different general tasks and splits them into held-in and held-out datasets. After tuning on the former, zero-shot performance is evaluated on the latter with unseen datasets or even unseen tasks. In contrast, the second setting is often observed in the evaluation of domain-specific tasks. For example, LLaVA [16] reports fine-tuned performance on ScienceQA [100]. LLaVA-Med [29] reports results on biomedical VQA [103].

The above evaluation methods are usually limited to a small range of selected tasks or datasets, lacking a comprehensive quantitative comparison. To this end, some efforts have endeavored to develop new benchmarks specially designed for MLLMs [104,105]. For example, Fu *et al.* [104] constructed a comprehensive evaluation benchmark named MME, which includes a total of 14 perception and cognition tasks. All instruction-answer pairs in MME are manually designed to avoid data leakage. MMBench [105] is a benchmark specifically designed for evaluating multiple dimensions of model capabilities, using ChatGPT to match open responses with predefined choices. Video-ChatGPT [87] and Video-Bench [106] focus on video domains and propose specialized benchmarks as well as evaluation tools for assessment.

### Open set

In contrast to the closed-set questions, the responses to open-set questions can be more flexible, where MLLMs usually play a chatbot role. Because the content of the chat can be arbitrary, it would be trickier to judge than the closed-ended output. The criterion can be classified into manual scoring, GPT scoring and case study approaches. Manual scoring requires humans to assess the generated responses. This kind of approach often involves handcrafted questions that are designed to assess specific dimensions. For example, mPLUG-Owl [107] collects a visually related evaluation set to judge capabilities like natural image, diagram and flowchart understanding. Similarly, GPT4Tools [90] builds two sets for the fine-tuning and zero-shot performance, respectively, and evaluates the responses in terms of thought, action, arguments and the whole.

Since manual assessment is labor intensive, some researchers have explored rating with GPT, namely, GPT scoring. This approach is often used to evaluate performance on multimodal dialogue. LLaVA [16] proposes to score the responses via text-only GPT-4 in terms of different aspects, such as helpfulness and accuracy. Specifically, 30 images are sampled from the COCO [108] validation set, each associated with a short question, a detailed question and a complex reasoning question via self-instruction on GPT-4. The answers generated by both the model and GPT-4 are sent to GPT-4 for comparison. Subsequent works follow this idea and prompt ChatGPT or GPT-4 to rate results [29] or judge which one is better [109].

A main issue of applying text-only GPT-4 for evaluation is that the judge is only based on translated text content, such as captions or bounding box coordinates, without accessing the image [29]. Thus, it may be questionable to set GPT-4 as the performance upper bound in this case. With the release of the vision interface of GPT, some works exploit the more advanced GPT-4V model to assess the performance of MLLMs. For example, Woodpecker [63] adopts the GPT-4V model to judge the response quality of model answers. The evaluation is expected to be more accurate than using text-only GPT-4 since GPT-4V has direct access to the image.

Since the benchmark evaluation is not comprehensive enough, a supplementary approach is to compare the different capabilities of MLLMs through case studies. For instance, some studies evaluate two typical advanced commercial-use models, GPT-4V and Gemini. Yang *et al.* [110] performed in-depth qualitative analysis on GPT-4V by crafting a series of samples across various domains and tasks, spanning from preliminary skills, such as caption and object counting, to complex tasks that require world knowledge and reasoning, such as joke understanding and indoor navigation as an embodied agent. Wen *et al.* [111] made a more focused evaluation of GPT-4V by designing samples targeting automatic driving scenarios. Fu *et al.* [112] carried out a comprehensive evaluation on Gemini-Pro by comparing the model against GPT-4V. The results suggest that GPT-4V and Gemini exhibit comparable visual reasoning abilities in spite of different response styles.

## EXTENSIONS

Recent studies have made significant strides in extending the capabilities of MLLMs, spanning from more potent foundational abilities to broader coverage of scenarios. We trace the principal development of MLLMs in this regard.

### Granularity support

To facilitate better interaction between agents and users, researchers have developed MLLMs with finer support of granularities in terms of model inputs and outputs. On the input side, models that support finer control from user prompts are developed progressively, evolving from image to region [24] and even pixels [25]. Specifically, Shikra [24] supports region-level input and understanding. Users may interact with the assistant more flexibly by referring to specific regions, which are represented in bounding boxes of natural language forms. Ferret [113] takes a step further and supports more flexible referring by devising a hybrid representation scheme. The model supports different forms of prompts, including point, box and sketch. Similarly, Osprey [25] supports point input by utilizing a segmentation model [10]. Aided by the exceptional capabilities of the pre-trained segmentation model, Osprey enables specifying a single entity or part of it with a single click. On the output side, grounding capabilities are improved in line with the development of input support. Shikra [24] supports response grounded in the image with box annotations, resulting in higher precision and finer referring experience. LISA [114] further supports mask-level understanding and reasoning, which makes pixel-level grounding possible.

### Modality support

Increased support for modalities is a tendency for MLLM studies. On the one hand, researchers have explored adapting MLLMs to support the input of more multimodal content, such as the three-dimensional point cloud [115]. On the other hand, MLLMs are also extended to generate responses of more modalities, such as image [116], audio [117] and video [118]. For example, NExT-GPT [119] proposes a framework that supports inputs and outputs of mixed modalities, specifically, combinations of text, image, audio and video, with the help of diffusion models [120] attached to the MLLM. The framework applies an encoder-decoder architecture and puts the LLM as a pivot for understanding and reasoning.

### Language support

Current models are predominantly unilingual, probably due to the fact that a high-quality non-English training corpus is scarce. Some works have been devoted to developing multilingual models so that a broader range of users can be covered. VisCPM [121] transfers model capabilities to the multilingual setting by designing a multi-stage training scheme. Specifically, the scheme takes English as a pivotal language, with an abundant training corpus. Utilizing a pre-trained bilingual LLM, the multimodal capabilities are transferred to Chinese by adding some translated samples during instruction tuning. Taking a similar approach, Qwen-VL [28] is developed from the bilingual LLM Qwen [48] and supports both Chinese and English. During pre-training, Chinese data are mixed into the training corpus to preserve the bilingual capabilities of the model, taking up 22.7% of the whole data volume.

### Scenario/task extension

Apart from developing common general-purpose assistants, some studies have focused on more specific scenarios where practical conditions should be considered, while others extend MLLMs to downstream tasks with specific expertise.

A typical tendency is to adapt MLLMs to more specific real-life scenarios. For example, some works develop agents that interact with the real world, e.g. user-friendly assistants specially designed for GUI, as exemplified by CogAgent [32], AppAgent [122] and Mobile-Agent [123]. Researchers also develop embodied agents [19,31] that can perform reasoning, navigation and manipulation in the real world, facilitating the development of automatic agents that can execute tasks for humans. In general, these assistants excel in planning and performing each step to fulfill tasks specified by users, acting as helpful agents for humans.

Another line is to augment MLLMs with specific skills for solving tasks in different domains, e.g. document understanding [30] and medical domains [29]. For document understanding, mPLUG-DocOwl [124] utilizes various forms of document-level data for tuning, resulting in an enhanced model in OCR-free document understanding. TextMonkey [30] incorporates multiple tasks related to document understanding to improve model performance. Similarly, MLLMs can also be trained to accommodate traditional vision tasks such as visual grounding [125,126]. Compared with traditional methods [13,127], MLLMs unify the I/O format and streamline the whole learning and inference process. Specifically, it is feasible to recast the grounding task into a conditioned box coordinate prediction task under a unified language modeling objective [24,28,52]. The model is trained to predict the coordinates of specified objects in the form of natural language. MLLMs can also be extended to medical domains by instilling specialized knowledge. For example, LLaVA-Med [29] develops assistants specialized in medical image understanding and question answering by injecting domain knowledge.

### Efficient MLLMs

Recently, using lightweight MLLMs for efficient deployment has gained increased popularity [128–130). These models are meticulously designed and optimized for more economical utilization or resource-limited scenarios without compromising too much on model performance.

From a model perspective, various techniques have been explored to facilitate efficient training and inference. For instance, MobileVLM [54] explores developing small-size variants of MLLMs for resource-limited scenarios. Some designs and techniques are utilized for deployment on mobile devices, such as LLMs of smaller size and quantization techniques to speed up computation. Similarly, MiniCPM-V [129] builds efficient MLLMs for end-side computation. A Q-Former [28] is adopted to cut down the number of visual tokens for each patch of the image.

From a data perspective, Bunny [130] comprehensively investigates efficient data selection and combination schemes for model training. The obtained models achieve performance on par with MLLMs of larger parameter sizes.

## MULTIMODAL HALLUCINATION

Multimodal hallucination refers to the phenomenon of responses generated by MLLMs being inconsistent with the image content [63]. The fundamental problem has received increased attention. In this section, we briefly introduce related concepts and research development.

### Preliminaries

Multimodal hallucinations can be categorized into three types [131].

- *Existence hallucination* is a common type, meaning that models incorrectly decide the existence of objects.
- *Attribute hallucination* means falsely describing the attributes of certain objects, e.g. failure to identify a dog’s color.
- *Relationship hallucination* is a more complex type of hallucination. It refers to false descriptions of relationships between objects, such as relative positions.

In what follows, we first introduce evaluation methods, which are useful to gauge the performance of methods for mitigating hallucinations. Then, we discuss mitigation methods of different kinds of approaches.

### Evaluation methods

CHAIR [132] is an early metric that evaluates hallucination levels in open-ended captions. The metric measures the proportion of sentences with hallucinated objects or the proportion of hallucinated objects in all the objects mentioned. In contrast, POPE [133] is a method that evaluates closed-set choices. Specifically, multiple prompts with binary choices are formulated, each querying if a specific object exists in the image. With a similar evaluation approach, MME [104] provides a more comprehensive evaluation, covering aspects of existence, count, position and color, as exemplified in [63].

Different from previous approaches that use matching mechanisms to detect hallucinations, some works explore automatic evaluation of text responses via models. For example, HaELM [134] proposes using LLMs as a judge to decide whether MLLMs’ captions are correct against reference captions. In view of the fact that text-only LLMs can only access limited image context and require reference annotations, Woodpecker [63] uses GPT-4V to directly assess model responses grounded in the image.

### Mitigation methods

According to high-level ideas for mitigating hallucinations, current methods can be roughly divided into three categories: pre-correction, in-process correction and post-correction.

*Pre-correction.* An intuitive solution for hallucination is to collect specialized data (e.g. negative data) and use the data for fine-tuning, thus achieving models with fewer hallucinations.

LRV-Instruction [135] introduces a visual instruction–tuning dataset to encourage faithful generation. Similarly, LLaVA-RLHF [96] collects human-preference pairs and fine-tunes models with reinforcement learning techniques.

*In-process correction.* Another line is to make improvements in architectural design or feature representation. These works try to explore the reasons for hallucinations and design remedies to mitigate them in the generation process. For example, HallE-Switch [131] introduces a continuous controlling factor to control the extent of imagination in model output during inference.

*Post-correction.* Different from previous paradigms, post-correction mitigates hallucinations in a post-remedy way. For example, Woodpecker [63] is a training-free framework for hallucination correction. Specifically, the method incorporates expert models to supplement contextual information of the image and crafts a pipeline to correct hallucinations step by step.

## EXTENDED TECHNIQUES

### Multimodal in-context learning

ICL is one of the important emergent abilities of LLMs. The essence of the technique is prompting the model with a few examples as guidance to make it easier for the model to answer the query. There are two good traits of ICL. (i) The crux of ICL is to learn from analogy [136], thus largely reducing the requirement of data samples. (ii) ICL is usually implemented in a training-free way [136] and can be flexibly integrated into various frameworks at inference time.

**Scheme 4.**
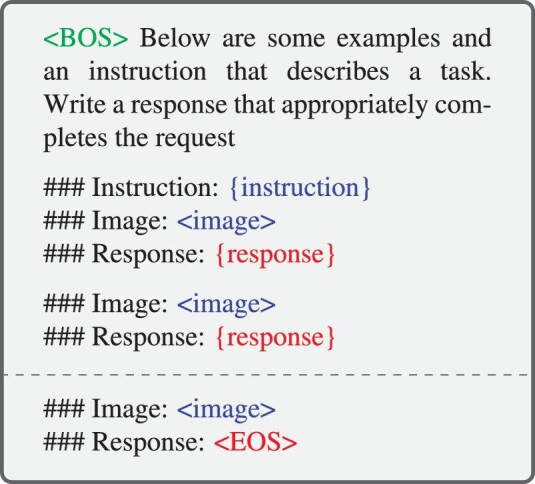
A simplified example of the template to structure an M-ICL query, adapted from [81]. For illustration, we list two in-context examples and a query divided by a dashed line. {instruction} and {response} are texts from the data sample. $< $image$> $ is a placeholder to represent the multimodal input (an image in this case). $< $BOS$> $ and $< $EOS$> $ are tokens denoting the start and the end of the input to the LLM, respectively.

In the context of the MLLM, ICL has been extended to more modalities, leading to multimodal in-context learning (M-ICL). At inference time, M-ICL can be implemented by adding a demonstration set, i.e. a set of in-context samples, to the original sample. In this case, the template can be extended, as illustrated in Scheme 4.

#### Improvement on ICL capabilities

Recently, a growing amount of work has focused on enhancing ICL performance under various scenarios. In this section, we trace the development of this field and summarize relevant works.

MIMIC-IT [137] combines in-context learning with instruction tuning by building an instruction dataset formatted with multimodal context. Some other works explore improving few-shot learning performance under specific settings. For example, Link-context learning [138] focuses on the causal relationships between demonstrations and queries, and casts a contrast training scheme by formulating positive and negative image-description pairs. Similarly, Yang *et al.* [139,140] explored different strategies to optimize demonstration configurations (selections or orderings of in-context samples) to achieve better few-shot performance.

#### Applications

In terms of applications in multimodality, M-ICL is mainly used in two scenarios: solving various visual reasoning tasks [141] and teaching LLMs to use external tools [142,143]. The former involves learning from several task-specific examples and generalizing to a new but similar question. In contrast, examples of tool usage are more fine grained, typically comprising a chain of steps to fulfill the task.

### Multimodal chain of thought

CoT is ‘a series of intermediate reasoning steps’ [5]. The technique has been proven to be effective in complex reasoning tasks. The main idea is to prompt LLMs to output not only the final answer, but also the reasoning process that leads to the answer, resembling the cognitive process of humans.

Inspired by the success in NLP realms, multiple works [144,145] have proposed to extend the technique to multimodal CoT (M-CoT). We first introduce different paradigms for acquiring the M-CoT ability. Then, we delineate more specific aspects of M-CoT, including the chain configuration and the pattern.

#### Learning paradigms

There are broadly three ways to acquire the M-CoT ability: through fine-tuning and training-free few- or zero-shot learning.

Intuitively, the fine-tuning approach often involves curating specific datasets for M-CoT learning. For example, Lu *et al.* [100] constructed a scientific question-answering dataset ScienceQA with lectures and explanations, which can serve as sources of learning CoT reasoning.

Compared with fine-tuning, few/zero-shot learning is more computationally efficient. The few-shot learning approach typically requires hand-crafted in-context examples to teach reasoning step by step. In contrast, the zero-shot learning approach directly prompts with designed instructions [144].

#### Chain configuration

Structure and length are two critical aspects of the reasoning chains. In terms of structure, current methods can be divided into single-chain [100] and tree-shape methods [146]. Chain length can be categorized into adaptive and predefined formations. The former configuration requires LLMs to decide when to halt the reasoning chains [100], while the latter setting stops the chains with a predefined length [147].

#### Generation patterns

We summarize the relevant works into an infilling-based pattern and a predicting-based pattern. Specifically, the infilling-based pattern demands deducing steps between surrounding context (previous and following steps) to fill the logical gaps [144]. In contrast, the predicting-based pattern requires extending the reasoning chains given conditions such as instructions and previous reasoning history [142].

### LLM-aided visual reasoning

#### Introduction

Inspired by the success of tool-augmented LLMs [148], some researchers have explored the possibilities of invoking external tools or vision foundation models for visual reasoning tasks. Taking LLMs as helpers with different roles, these works build task-specific or general-purpose visual reasoning systems.

Compared with conventional visual reasoning models, these works manifest several good traits.

- *Strong generalization abilities:* equipped with rich open-world knowledge learned from large-scale pre-training, these systems can easily generalize to unseen objects or concepts with remarkable zero/few-shot performance [149].
- *Emergent abilities:* aided by the strong reasoning abilities of LLMs, these systems can perform complex tasks, e.g. understanding the deep meaning of an image [18].
- *Better interactivity and control:* traditional models typically allow a limited set of control mechanisms, while LLM-based systems enable finer control in a user-friendly interface (e.g. click and natural language queries) [150].

For this part, we start by introducing different training paradigms employed in the construction of LLM-aided visual reasoning systems. Then, we delve into the primary roles that LLMs play within these systems.

#### Training paradigms

According to training paradigms, LLM-aided visual reasoning systems can be divided into two types: training-free and fine-tuning.

*Training-free.* With abundant prior knowledge stored in pre-trained LLMs, an intuitive and simple way is to freeze pre-trained models and directly prompt LLMs to fulfill various needs. According to the setting, the reasoning systems can be further categorized into few-shot models [142] and zero-shot models [150].

*Fine-tuning.* Some works adopt further fine-tuning to improve the planning abilities with respect to tool usage [90] or to improve localization capabilities [114] of the system. For example, GPT4Tools [90] collects a tool-related instruction dataset to fine-tune the model.

#### Functions

Regarding what roles LLMs exactly play in LLM-aided visual reasoning systems, existing related works are divided into three types:

- the LLM as a controller;
- the LLM as a decision maker;
- the LLM as a semantics refiner.

We delineate how LLMs serve these roles in the following.

*The LLM as a controller.* In this case, LLMs act as a central controller that breaks down a complex task into simpler sub-tasks/steps and assigns these tasks to appropriate tools/modules. Specifically, LLMs are prompted explicitly to output task planning [151] or, more directly, the modules to call [90,142,143]. For example, VisProg [143] prompts GPT-3 to output a visual program, where each program line invokes a module to perform a sub-task.

*The LLM as a decision maker.* In this case, complex tasks are solved in a multi-round manner, often in an iterative way [152]. Decision-makers often summarize the context to decide whether to finish the task and organize the answer in a user-friendly way.

*The LLM as a semantics refiner.* When the LLM is used as a semantics refiner, researchers mainly utilize its rich linguistic and semantic knowledge. Specifically, LLMs are often instructed to integrate information into fluent natural language sentences [153] or generate texts according to different specific needs [149,150,154].

## CHALLENGES AND FUTURE DIRECTIONS

The development of MLLMs is still in a rudimentary stage and thus leaves much room for improvement, which we summarize below.

- Current MLLMs are limited in processing multimodal information of long context. This restricts the development of advanced models with more multimodal tokens, e.g. long-video understanding and long documents interleaved with images and text.
- MLLMs should be upgraded to follow more complicated instructions. For example, a mainstream approach to generating high-quality question-answer pair data is still prompting closed-source GPT-4V because of its advanced instruction-following capabilities, while other models generally fail to achieve such goals.
- There is still a large space for improvement in techniques like M-ICL and M-CoT. Current research on the two techniques is still rudimentary, and the related capabilities of MLLMs are still weak. Therefore, explorations on the underlying mechanisms and potential improvements are promising.
- Developing embodied agents based on MLLMs is a heated topic. It would be meaningful to develop such agents that can interact with the real world. Such endeavors require models with critical capabilities, including perception, reasoning, planning and execution.
- Safety issues: similar to LLMs, MLLMs can be vulnerable to crafted attacks. In other words, MLLMs can be misled to output biased or undesirable responses. Thus, improving model safety will be an important research topic.
- Interdisciplinary research: given the strong generalization capabilities and abundant pre-trained knowledge of MLLMs, a promising research direction could be utilizing MLLMs to boost research fields of natural sciences, e.g. leveraging MLLMs for analysis of medical images or remote sensing images. To achieve this goal, injecting domain-specific multimodal knowledge into MLLMs might be necessary.

## CONCLUSION

In this paper, we review the existing MLLM literature and offer a broad view of its main directions, including the basic recipe and related extensions. Moreover, we underscore the current research gaps that need to be filled and point out some promising research directions. We hope that this survey can offer readers a clear picture of the current progress of the MLLM and inspire more relevant works. In light of the fact that the era of the MLLM has only just begun, we will keep updating this survey and hope that it can inspire more research. An associated GitHub link collecting the latest papers is available at: https://github.com/BradyFU/Awesome-Multimodal-Large-Language-Models.
